# Zervimesine (CT1812) Treatment Benefits Patients with Lower Baseline Plasma p‐tau217 Across the Mild‐to‐Moderate AD Spectrum

**DOI:** 10.1002/alz70859_106858

**Published:** 2025-12-26

**Authors:** Michael Grundman, Susan Catalano, Mary E. Hamby, Theresa Devins, Jennifer Iaci, Anthony O Caggiano

**Affiliations:** ^1^ Global R&D Partners, LLC, La Jolla, CA USA; ^2^ Capsida Therapeutics, Thousand Oaks, CA USA; ^3^ Cognition Therapeutics, Inc., Pittsburgh, PA USA; ^4^ Cognition Therapeutics, Purchase, NY USA; ^5^ Cognition Therapeutics, Inc., Purchase, NY USA

## Abstract

**Background:**

Zervimesine (CT1812) is an experimental oral, small‐molecule drug candidate in development for Alzheimer’s disease (AD) and dementia with Lewy bodies. Zervimesine is designed to protect neuronal synapses by preventing the binding of amyloid beta (Aβ) oligomers.

**Method:**

The Phase 2 COG0201 ‘SHINE’ clinical trial measured tolerability and clinical effects of zervimesine in adults with amyloid‐positive mild‐to‐moderate AD (MMSE 18 to 26). The study enrolled 153 individuals at 30 U.S. and international sites. Participants were randomized 1:1:1 to receive placebo or one of two doses (100 or 300mg) of oral zervimesine daily for 26 weeks. Patients were assessed using a traditional battery of symptomatic assessments (ADAS‐Cog, MMSE, ADSC‐ADL and CGIC). A pre‐specified analysis evaluated treatment effect relative to placebo based on median plasma p‐tau217 value.

**Result:**

In the full mITT population in SHINE, zervimesine‐treated participants (n=101) declined 39% less on ADAS‐Cog 11 and 70% less on MMSE relative to placebo (n=49). Participants with plasma p‐tau217 levels below the median of 1.0 pg/mL had an even more robust response. Zervimesine‐treated patients with sub‐median levels of plasma p‐tau217 (n=45) experienced 95% less cognitive decline on ADAS‐Cog 11 and 108% less decline on MMSE vs placebo (n=24). We will present for the first time at AAIC the distribution of plasma p‐tau217 by MMSE severity in SHINE and demonstrate that this enhanced response was similar for patients with MMSE 22‐26 and MMSE 18‐21, encompassing the full range of baseline severity in this study.

**Conclusion:**

Results from the SHINE study provide compelling evidence that zervimesine has the potential to slow cognitive decline in patients with AD. Participants with lower disease burden, as measured by plasma p‐tau217 experienced a more pronounced treatment effect. Importantly, this benefit was observed in people across the mild‐to‐moderate MMSE spectrum. While preliminary, these results suggest that having a lower disease burden as measured by plasma p‐tau217 may provide a therapeutic window to still treat patients in the moderate MMSE range.

SHINE was supported by a grant from the National Institute of Aging (R01AG058660).

